# Effects of oral versus transdermal menopausal hormone treatments on self-reported sleep domains and their association with vasomotor symptoms in recently menopausal women enrolled in the Kronos Early Estrogen Prevention Study (KEEPS)

**DOI:** 10.1097/GME.0000000000000971

**Published:** 2018-01-26

**Authors:** Dahima Cintron, Brian D. Lahr, Kent R. Bailey, Nanette Santoro, Robin Lloyd, JoAnn E. Manson, Genevieve Neal-Perry, Lubna Pal, Hugh S. Taylor, Whitney Wharton, Fredrick Naftolin, S. Mitchell Harman, Virginia M. Miller

**Affiliations:** 1 Mayo Clinic Graduate School, Mayo Clinic Rochester, MN; 2 Department of Health Sciences Research Division of Biomedical Statistics and Informatics, Mayo Clinic Rochester, MN; 3 Department of Obstetrics and Gynecology, University of Colorado at Denver, Aurora, CO; 4 Department of Internal Medicine, Mayo Clinic, Rochester, MN; 5 Department of Medicine, Brigham and Women's Hospital, Harvard Medical School, Boston, MA; 6 Department of Obstetrics and Gynecology, University of Washington, Seattle, WA; 7 Department of Obstetrics, Gynecology and Reproductive Sciences, Yale University School of Medicine, New Haven, CT; 8 Department of Neurology, Emory University, Atlanta, GA; 9 NYU Interdisciplinary Program in Menopause Medicine, New York University, New York, NY; 10 Kronos Longevity Research Institute and the Phoenix Veterans Administration Health Care System, Phoenix, AZ; 11 Department of Surgery and Department of Physiology and Biomedical Engineering, Mayo Clinic, Rochester, MN.

**Keywords:** Conjugated equine estrogens, Estradiol, Hot flashes, Night sweats, Pittsburgh Sleep Quality Index, Vasomotor symptoms

## Abstract

**Objective:**

This study determined whether two different formulations of hormone therapy (HT): oral conjugated equine estrogens (o-CEE; 0.45 mg/d, n = 209), transdermal 17β-estradiol (t-E2; 50 μg/d, n = 201) plus cyclic progesterone (Prometrium, 200 mg) or placebo (PBO, n = 243) affected sleep domains in participants of the Kronos Early Estrogen Prevention Study.

**Methods:**

Participants completed the Pittsburgh Sleep Quality Index at baseline and during the intervention at 6, 18, 36, and 48 months. Global sleep quality and individual sleep domain scores were compared between treatments using analysis of covariance, and correlated with vasomotor symptom (VMS) scores using Spearman correlation coefficients.

**Results:**

Global Pittsburgh Sleep Quality Index scores (mean 6.3; 24% with score >8) were similar across groups at baseline and were reduced (improved sleep quality) by both HT (average change −1.27 [o-CEE] and −1.32 [t-E2]) when compared with PBO (−0.60; *P* = 0.001 [o-CEE vs PBO] and *P* = 0.002 [t-E2 vs PBO]). Domain scores for sleep satisfaction and latency improved with both HT. The domain score for sleep disturbances improved more with t-E2 than o-CEE or PBO. Global sleep scores significantly correlated with VMS severity (*r*_s_ = 0.170, *P* < 0.001 for hot flashes; *r*_s_ = 0.177, *P* < 0.001 for night sweats). Change in scores for all domains except sleep latency and sleep efficiency correlated with change in severity of VMS.

**Conclusions:**

Poor sleep quality is common in recently menopausal women. Sleep quality improved with both HT formulations. The relationship of VMS with domains of sleep suggests that assessing severity of symptoms and domains of sleep may help direct therapy to improve sleep for postmenopausal women.

Chronic sleep deprivation is associated with both short and long-term health consequences including fatigue, impaired memory, and increased risk for cardiovascular disease and diabetes.^1^ Forty to sixty per cent of women report problems sleeping during the perimenopause and early menopause.^2^ Data from The North American Menopause Society posits that about 73% of postmenopausal women report vasomotor symptoms (VMS) (ie, hot flashes and night sweats). VMS may also be a risk factor for future cardiovascular disease.^3,4^ Hormone therapy (HT) is an effective treatment for common VMS, and also for alleviation of some sleep problems during menopause.^5-7^ Many studies have evaluated the relationships among HT, sleep, and VMS in perimenopausal women.^8-10^ It is difficult overall to disentangle age-related worsening of sleep from menopause-driven change in sleep. In one 14-year longitudinal examination of the menopausal transition, no strong relationship between menopause and worsening sleep was observed. However, a small group of “at-risk” women were identified who had poor sleep at baseline, and who reported VMS.^11^ This group of women was most likely to have acute worsening of their sleep as they traversed the menopause. In agreement with this body of research, a systematic appraisal of the literature suggests that only a subgroup of women with concomitant VMS may derive a modest improvement in sleep from HT.^12^ Therefore, questions remain regarding the association of domains of sleep (sleep disturbances compared with overall sleep quality) in relationship to VMS in menopause and HT.^13,14^

There are three major difficulties in assessing the relationships between VMS, HT, and sleep. First, a variety of HT formulations, doses, and routes of administration are used to treat menopausal symptoms, making comparisons among studies difficult. No studies have compared the effectiveness of two types of HT on their relationship to alleviation of VMS and domains of sleep. The second difficulty emanates from a heterogeneity in the tools used to evaluate and characterize sleep.^15^ Clinically, poor sleep quality is defined as perceived sleep problems that are bothersome, but do not meet criteria for a clinical sleep disorder.^16,17^ However, poor sleep quality may be comprised of different factors, or domains, for different individuals; therefore, a standard tool that measures sleep through multiple domains allows for optimal characterization of sleep quality^15^ and may provide a better insight into the associations between alleviation of VMS and sleep in menopausal women. A third difficulty is that self-reported changes in VMS may not accurately reflect their frequency, duration, and severity.^18-20^

The purpose of this study was to evaluate the effects of two formulations of HT on self-reported sleep quality and sleep domains using the broadly accepted Pittsburgh Sleep Quality Index (PSQI); and to assess the relationship between the changes in global sleep quality, and the domains of sleep with VMS for each HT formulation compared with placebo (PBO) in recently menopausal women enrolled in the Kronos Early Estrogen Prevention Study (KEEPS).

## METHODS

### Participants

The KEEPS (NCT00154180)—a randomized, double-blind, PBO-controlled multisite trial—enrolled women from the communities surrounding each recruitment site who were between the ages of 42 and 58 years, between 6 and 36 months since their last menses, and had serum follicle-stimulating hormone level ≥35 mIU/mL and/or estradiol level <40 pg/mL. Women were excluded if they had a coronary artery calcium score of >50 Agatston Units, history of cardiovascular disease, body mass index (BMI) >35 kg/m^2^, uncontrolled hypertension (systolic blood pressure >150 mm Hg and/or diastolic blood pressure >95 mm Hg), low-density lipoprotein cholesterol (LDL) >190 mg/dL, triglycerides (Tg) >400 mg/dL, fasting blood glucose >126 mg/dL (or history of diabetes), current or recent (6 months) use of cholesterol-lowering medications (statins, fibrate, or >500 mg/d niacin), if they reported smoking more than 10 cigarettes per day, or had a diagnosis of clinical depression. The study was approved by Institutional Review Boards at each participating institution. All participants were recruited and all gave written informed consent. The study design and methods have been described in detail elsewhere.^21^

### Study design

Participants (n = 727) were randomized (4:4:5 ratio) to either oral conjugated equine estrogens (o-CEE; Premarin, 0.45 mg/d) plus a PBO transdermal patch (n = 230), transdermal 17β-estradiol (t-E2; Climara 50 μg/d) plus a PBO pill (n = 222), or PBO pills and patch (n = 275). Women in the active treatment groups also received oral micronized progesterone (Prometrium, 200 mg) for the first 12 days of each month, whereas women in the PBO group received a PBO capsule for the first 12 days of each month.

The randomization sequence generation was done using a random number table. Study drugs were supplied to the clinical sites identified only by the participant's ID number, with both research participants and investigators blinded to treatment. Treatment was given for 4 years. Participants at all sites were invited to complete the PSQI before randomization (baseline) and then at 6, 18, 36, and 48-month study visits, which coincided with cognitive testing (NCT00623311).^22,23^ Changes in the domains of sleep were not prespecified outcomes from the main KEEPS protocol, but including the PSQI questionnaire to the study visits was approved as an ancillary study to the main protocol.

### Outcomes

#### PSQI for sleep parameters

The PSQI is a brief self-report, nine multi-item questionnaire, assessing seven domains of sleep quality: sleep satisfaction, sleep latency, sleep duration, habitual sleep efficiency, sleep disturbances, use of sleeping medications, and staying awake during daytime activities (daytime dysfunction).^24^ The PSQI has strong reliability and validity, in both clinical and nonclinical samples.^25^

Each of the seven domains of sleep is clinically relevant for evaluating sleep and is scored from a range of 0 to 3 points: 0 = no difficulty, 3 = severe difficulty.^24^ The global sleep score represents a summation of the individual sleep domain scores 0 to 21. Poor sleep quality was defined by a PSQI global score >8, which is the clinical threshold for requiring complete somnological evaluation.^26^ Analyses were performed on PSQI global scores and on six out of seven individual domains: sleep satisfaction, sleep latency, habitual sleep efficiency, sleep disturbances, sleep duration, and daytime dysfunction. Use of medication was not included in the present analysis, because the mean baseline score for this domain was 0.61, with 69% of the participants (71% in PBO, 67% in t-E2, and 68% in o-CEE) reporting not taking medications for sleep in the previous month. Caffeine intake was not recorded.

#### Vasomotor symptom scale

To assess the burden of hot flashes and night sweats over time, participants completed a questionnaire reporting symptoms of hot flashes and night sweats over the past 3 months, ranking symptoms on a 4-point Likert scale as none (0), mild (1), moderate (2), or severe (3). As reported previously,^27^ these symptoms were evaluated retrospectively at baseline and annually during the study period, with an average score calculated and compared with baseline for each symptom.

### Statistical analysis

Descriptive statistics on baseline characteristics are reported as number and percentage for categorical variables (which were dichotomized), and as mean and standard deviation (SD) for continuous variables. Baseline comparisons between randomized treatment groups were performed using Pearson's chi-square test or analysis of variance, as appropriate. The study outcomes of interest included the PSQI global score (0-21) and six preselected domain scores (0-3), and also two menopausal symptom severity scores (0-3). To describe the treatment response for each outcome treated as a continuous variable, repeated measurements were reduced into a single measure of response by computing the average score per participant during the treatment period and subtracting their baseline value. Since changes in the outcome variables were generally observed early at 6-month assessment and sustained through the study visits at 48 months, it was reasoned that the average change provides an adequate summary of the response and makes better use of available information than the change computed at a single follow-up time. To augment the description of PSQI global scores in this study, the measured continuous scale was also dichotomized at a clinically relevant threshold score of >8 to indicate poor sleep quality.^28^

Formal comparison of the change in response between treatments was performed via analysis of covariance (ANCOVA) using all available follow-up measurements, with use of the generalized estimating equation (GEE) method to account for correlation among the repeated measures on participants. One ANCOVA model was fit to each outcome variable, with the follow-up score included as the dependent variable, treatment group as the primary independent variable, and baseline score as the covariate. Study site was included in the models as an additional adjusting covariate. Race was not included as a covariate as the majority of participants were white by self-report and ancestry genotype.^29,30^ To avoid deletion of a small subset of individuals lacking a baseline PSQI measurement (n = 38), the mean baseline value of the combined sample was imputed for these individuals. Each ANCOVA model yielded a least squares mean estimate of the baseline-adjusted change within treatment group, presented as mean and 95% confidence interval (CI). Treatment group differences in these adjusted changes were assessed by testing the group term in the model for significance, using an omnibus 2 degree of freedom test; individual pair-wise comparisons were carried out only if the overall test revealed evidence of any statistically significant difference (if *P* ≤ 0.05). An analogous model based on the GEE formulation of logistic regression was used for the binary PSQI outcome to test whether the change in rate of poor sleep quality differed between treatment groups after adjustment for baseline rate.

Secondary analyses were performed to assess the relation between changes in PSQI scores (global and individual domain scores) and changes in the menopausal symptom scores based on the partial Spearman's rank correlation coefficient (*r*_s_), which were adjusted for treatment group. The same method was used to perform a post hoc subgroup analysis to assess the correlation between changes in sleep quality and changes in menopausal symptoms in women who reported moderate to severe hot flashes and night sweats (score 2 or 3 on VMS scale) at baseline. All analyses were carried out in SAS software version 9.4 (SAS Institute Inc., Cary, NC).

## RESULTS

**FIG. 1:**
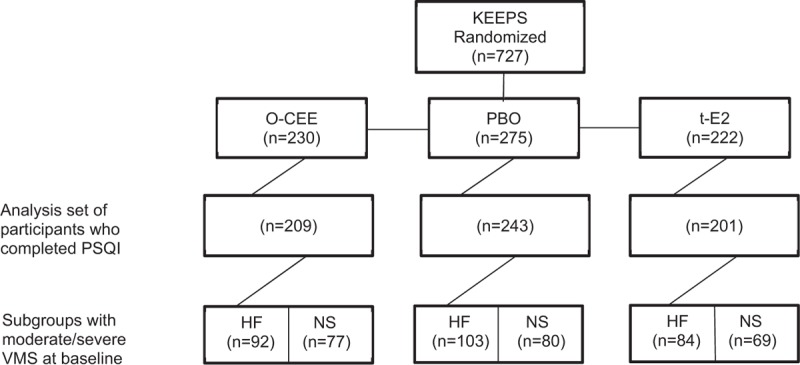
Flow diagram of the Kronos Early Estrogen Prevention Study (KEEPS) participants included in study analysis. HF, hot flashes; NS, night sweats; o-CEE, oral conjugated equine estrogen; PBO, placebo; PSQI, Pittsburgh Sleep Quality Index; t-E2, transdermal estradiol; VMS, moderate to severe vasomotor symptoms at baseline subgroup. It should be noted that the numbers for women with hot flashes and night sweats are not mutually exclusive.

In all, 727 women were enrolled in the KEEPS, with 275 randomly assigned to PBO, 230 to oral (o-CEE), and 222 to transdermal (t-E2) treatments. Baseline clinical parameters and menopausal symptoms (Table 1), and also education status, incomes, marital status, and number of term pregnancies^27,31^ were similar among treatment assignments and were not different from women excluded from the main KEEPS protocol.^29^ For the primary analysis, the effects of treatment on aspects of sleep were assessed on 653 (90%) participants who completed the PSQI at one or more study visits during the 4-year intervention period (Fig. 1). To examine the potential for selection bias, the 653 PSQI respondents were compared with the remaining 74 KEEPS participants who did not complete the PSQI, and no significant differences were found in baseline clinical parameters (results not shown).

**TABLE 1:** Comparisons of baseline characteristics among treatment groups for total KEEPS

Variable	n	No. (%) missing	PBO (n = 275)	t-E2 (n = 222)	o-CEE (n = 230)	*P*
Age, y	726	1 (<1%)	52.5 ± 2.5	52.7 ± 2.6	52.8 ± 2.6	0.394
White, by self-report	690	37 (5%)	210 (80%)	169 (82%)	176 (80%)	0.863
Weight, kg	727	0	71.1 ± 12.2	69.9 ± 12.0	69.7 ± 11.9	0.322
Height, cm	727	0	164.1 ± 6.2	163.9 ± 6.1	163.6 ± 6.3	0.748
Body mass index, kg/m^2^	727	0	26.4 ± 4.3	26.0 ± 4.4	26.0 ± 4.3	0.502
Waist circumference, cm	712	15 (2%)	84.9 ± 12.0	83.8 ± 11.8	84.2 ± 11.3	0.566
Systolic blood pressure, mm Hg	727	0	119.8 ± 14.4	117.3 ± 15.8	118.9 ± 14.5	0.158
Diastolic blood pressure, mm Hg	727	0	75.3 ± 9.5	74.0 ± 9.7	75.2 ± 8.3	0.237
Total cholesterol, mg/dL	727	0	216.7 ± 30.4	215.2 ± 33.6	214.7 ± 32.1	0.759
Low-density cholesterol level, mg/dL	727	0	130.5 ± 29.3	127.8 ± 31.1	127.5 ± 27.9	0.459
High-density cholesterol level, mg/dL	727	0	63.8 ± 16.2	66.2 ± 18.5	66.4 ± 18.0	0.176
Triglyceride level, mg/dL^*a*^	727	0	92.7 ± 51.5	89.0 ± 45.6	89.3 ± 52.0	0.658
Menopausal symptoms						
Hot flash symptoms by history^*b*^	727	0	126 (46%)	92 (41%)	100 (43%)	0.617
Night sweats by history^*b*^	727	0	99 (36%)	72 (32%)	83 (36%)	0.643
Mood swings^*b*^	727	0	43 (16%)	38 (17%)	34 (15%)	0.789
Depression^*b*^	727	0	21 (8%)	19 (9%)	23 (10%)	0.641
Trouble sleeping^*b*^	727	0	93 (34%)	78 (35%)	66 (29%)	0.297
Irritability^*b*^	727	0	42 (15%)	42 (19%)	39 (17%)	0.559

KEEPS, the Kronos Early Estrogen Prevention Study; o-CEE, oral conjugated equine estrogen; PBO, placebo; t-E2, transdermal estradiol.

^*a*^Variable was transformed to the natural logarithmic scale before performing the statistical testing so that the test assumptions were not violated.

^*b*^Severity scales for menopausal symptoms were dichotomized as binary to indicate a response of either “moderate” or “severe.”

### PSQI global score

At baseline, PSQI global score did not differ significantly among treatment groups (Table 2). Overall, 24% of participants (22% PBO, 21% t-E2, and 28% o-CEE) had poor sleep quality (global scores >8) at study entry. Compared with baseline values, each of the two HT groups and the PBO group showed a reduction in average PSQI global scores over 4 years of treatment (each *P* < 0.001). The average reduction in the global score was similar between the two hormone-treated groups (average change of −1.27 [o-CEE] and −1.32 [t-E2] points), and both were significantly greater than that in the PBO group (−0.60 points; *P* = 0.001 [o-CEE vs PBO] and *P* = 0.002 [t-E2 vs PBO]; Table 2). Similarly, the percentage of women with poor sleep quality (PSQI global score >8) decreased with t-E2 (from 21% to 9%; *P* < 0.001) and o-CEE (from 28% to 16%; *P* < 0.001), with a smaller decline with PBO (from 22% to 17%; *P* = 0.06). When compared with PBO, the reduction in the percentage of women with poor sleep quality (ie, the number of women with improved sleep quality) was significantly greater in the t-E2 group (*P* = 0.003) and modestly but not significantly greater in the o-CEE group (*P* = 0.07).

**TABLE 2:** PSQI global and individual domain scores

Measurement	Placebo (PBO)	Transdermal (t-E2)	Oral (o-CEE)	Overall *P*	PBO/t-E2	Contrasts PBO/o-CEE	t-E2/o-CEE
PSQI score and subscales (n = 653)							
Global score							
Baseline score^*a*^	6.17 ± 3.06	6.10 ± 3.12	6.57 ± 3.36				
Mean follow-up score	5.69 ± 2.95	4.92 ± 2.63	5.23 ± 2.71				
Mean change (95% CI)^*b*^	−0.60 (−0.89, −0.31)^*c*^	−1.32 (−1.64, −1.00)^*c*^	−1.27 (−1.58, −0.95)^*c*^	0.001	0.002	0.001	0.975
Satisfaction/quality							
Baseline score^*a*^	1.12 ± 0.66	1.17 ± 0.75	1.20 ± 0.72				
Mean follow-up score	0.97 ± 0.55	0.85 ± 0.57	0.84 ± 0.55				
Mean change (95% CI)^*b*^	−0.19 (−0.25, −0.12)^*c*^	−0.32 (−0.39, −0.25)^*c*^	−0.34 (−0.41, −0.27)^*c*^	0.001	0.008	<0.001	0.449
Sleep latency							
Baseline score^*a*^	1.02 ± 0.92	0.95 ± 0.88	1.06 ± 0.93				
Mean follow-up score	0.97 ± 0.79	0.74 ± 0.70	0.79 ± 0.73				
Mean change (95% CI)^*b*^	−0.08 (−0.15, 0.00)	−0.27 (−0.36, −0.19)^*c*^	−0.28 (−0.36, −0.19)^*c*^	<0.001	0.002	<0.001	0.822
Sleep efficiency							
Baseline score^*a*^	0.46 ± 0.78	0.45 ± 0.72	0.61 ± 0.91				
Mean follow-up score	0.40 ± 0.64	0.35 ± 0.47	0.39 ± 0.64				
Mean change (95% CI)^*b*^	−0.10 (−0.16, −0.03)^*c*^	−0.15 (−0.23, −0.08)^*c*^	−0.16 (−0.24, −0.09)^*c*^	0.451			
Sleep disturbances							
Baseline score^*a*^	1.52 ± 0.54	1.44 ± 0.61	1.52 ± 0.60				
Mean follow-up score	1.43 ± 0.47	1.26 ± 0.41	1.39 ± 0.47				
Mean change (95% CI)^*b*^	−0.08 (−0.13, −0.03)^*c*^	−0.22 (−0.27, −0.16)^*c*^	−0.12 (−0.18, −0.07)^*c*^	0.001	<0.001	0.166	0.029
Sleep duration							
Baseline score^*a*^	0.54 ± 0.68	0.51 ± 0.70	0.57 ± 0.77				
Mean follow-up score	0.47 ± 0.64	0.43 ± 0.60	0.41 ± 0.58				
Mean change (95% CI)^*b*^	−0.08 (−0.14, −0.01)^*c*^	−0.10 (−0.18, −0.03)^*c*^	−0.15 (−0.22, −0.07)^*c*^	0.377			
Daytime dysfunction							
Baseline score^*a*^	0.91 ± 0.75	0.89 ± 0.70	0.84 ± 0.68				
Mean follow-up score	0.79 ± 0.59	0.71 ± 0.58	0.73 ± 0.55				
Mean change (95% CI)^*b*^	−0.11 (−0.17, −0.05)^*c*^	−0.19 (−0.26, −0.12)^*c*^	−0.14 (−0.21, −0.08)^*c*^	0.258			
Vasomotor symptom scores (n = 662)							
Hot flashes severity							
Baseline score^*a*^	1.39 ± 0.79	1.38 ± 0.82	1.37 ± 0.88				
Mean follow-up score	0.89 ± 0.68	0.44 ± 0.60	0.39 ± 0.46				
Mean change (95% CI)^*b*^	−0.48 (−0.55, −0.42)^*c*^	−0.93 (−1.00, −0.85)^*c*^	−0.98 (−1.05, −0.90)^*c*^	<0.001	<0.001	<0.001	0.343
Night sweats severity							
Baseline score^*a*^	1.08 ± 0.92	1.12 ± 0.95	1.08 ± 0.98				
Mean follow-up score	0.62 ± 0.67	0.33 ± 0.54	0.33 ± 0.46				
Mean change (95% CI)^*b*^	−0.47 (−0.54, −0.41)^*c*^	−0.76 (−0.83, −0.69)^*c*^	−0.76 (−0.83, −0.69)^*c*^	<0.001	<0.001	<0.001	0.919

PSQI results are reported on a total of 653 respondents (n = 243 [PBO], 201 [t-E2], 209 [o-CEE]), whereas vasomotor symptom results are presented on 662 respondents (n = 243 [PBO], 205 [t-E2], 214 [o-CEE]); to describe the treatment responses, repeated measurements were summarized by the average score per woman during the treatment period, whereas comparisons between treatments utilized all serial data points available via repeated measures analysis (see “Methods” section for details). Treatment groups were tested for a difference in response in ANCOVA models that included site and baseline score as covariates, with the GEE method used to account for within-participant correlation from repeated measurements. From each ANCOVA, the three-level treatment effect was screened for significance and only when such evidence was revealed did we proceed with post hoc testing of pair-wise group differences. CI, confidence interval; o-CEE, oral conjugated equine estrogen; PBO, placebo; PSQI, Pittsburgh Sleep Quality Index; t-E2, transdermal estradiol.

^*a*^There were no significant differences in baseline scores between treatments (*P* > 0.05 from analysis of variance [ANOVA]). To avoid case-wise deletion in analyses of treatment responses, the mean baseline estimate of each PSQI scale was imputed on n = 38 women who lacked a baseline measurement.

^*b*^Baseline-adjusted mean change over follow-up assessments was estimated via analysis of covariance [ANCOVA] modeling, and is reported as mean and 95% confidence interval.

^*c*^Indicates that the baseline-adjusted change within corresponding group was statistically significant (*P* < 0.05).

### PSQI individual domains score

**FIG. 2:**
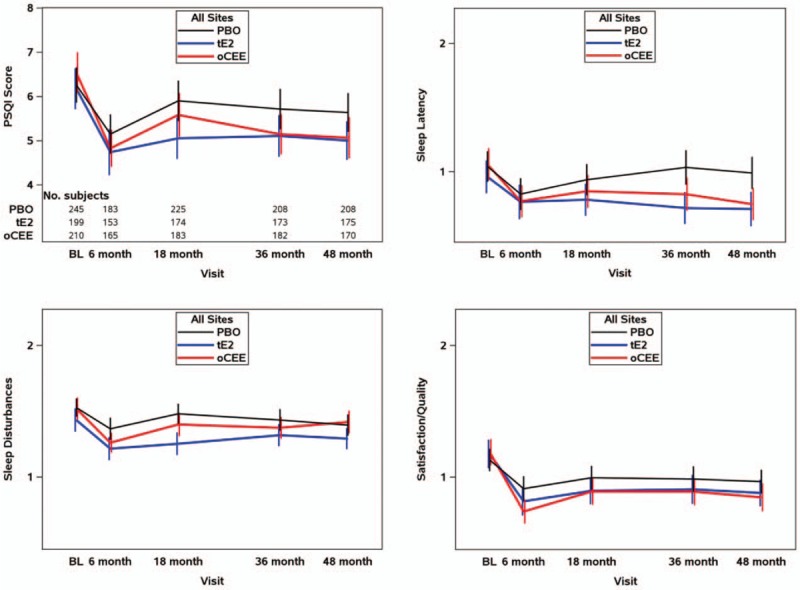
Longitudinal analysis for overall Pittsburgh Sleep Quality Index (PSQI) score and three domains of sleep. Line graphs show mean and 95% confidence intervals at each study visit to illustrate trends by treatment for the pooled set of participants These values are based on a total of 653 respondents (n = 243 [PBO], 201 [t-E2], and 209 [o-CEE]) with at least one survey during study follow-up, of whom 85% (86% [PBO], 87% [t-E2], and 81% [o-CEE]) contributed a score at the end-of-study 48-month visit. These graphs demonstrated significantly greater improvement (decrease in scores) in each of the two hormone-treated groups relative to those treated with PBO for global PSQI score, domains for sleep latency and sleep satisfaction. For the domain of sleep disturbance, only t-E2 showed a significantly greater improvement (decrease in score) than PBO, and was also the only scale to show a significant difference between the hormone treatment groups (greater improvement in those randomized to t-E2 than to o-CEE). o-CEE, oral conjugated equine estrogen; PBO, placebo; t-E2, transdermal estradiol.

Compared with baseline scores, significant improvements in all six domain scores were observed during treatment within each group, with the exception of sleep latency in the PBO group (*P* = 0.06; Table 2). The average improvement in score differed between treatments for three out of the six domains (sleep satisfaction, sleep latency, and sleep disturbances), with each of these three domains showing significantly greater improvement in one or both of the HT groups when compared with PBO **(**Fig. 2**)**. Among the three domains, only sleep disturbances showed a statistically significant difference between the two HT formulations (*P* = 0.029), with t-E2 improving more during follow-up than o-CEE (Table 2). There were no significant differences between groups for the changes in domain scores pertaining to sleep efficiency (*P* = 0.45), sleep duration (*P* = 0.38), or daytime dysfunction (*P* = 0.26) across follow-up.

### Association with VMS

A total of 662 women providing responses of VMS severity during the course of treatment were retained in the corresponding treatment comparisons. As reported previously for the entire KEEPS cohort,^27^ the average severity scores of hot flashes and night sweats were significantly reduced by both HT formulations when compared with PBO (*P* < 0.001 for both), with no difference between the o-CEE and t-E2 groups (*P* = 0.343 and *P* = 0.919, respectively; Table 2). For the 646 participants for whom both PSQI and VMS data were available, there were positive associations between the average change in global PSQI score and the average changes in severity of hot flashes (*r*_s_ = 0.170, *P* < 0.001) and night sweats (*r*_s_ = 0.177, *P* < 0.001; Table 3). Among the correlations assessed between changes in the individual sleep domain scores and changes in scores for hot flashes, all domains except sleep latency and sleep efficiency correlated positively with change in severity. In addition to the domains of sleep latency and sleep efficiency, the domain of sleep duration also did not correlate with changes in night sweats.

**TABLE 3:** Correlation (*r*_s_) of average change in PSQI scores with average change in VMS scores

	Overall analysis		Subgroup analysis women with moderate/severe symptoms at baseline	
PSQI measure	Hot flashes (n = 646)	Night sweats (n = 646)	Hot flashes (n = 279)	Night sweats (n = 226)
Global score	0.170 (*P* < 0.001)	0.177 (*P* < 0.001)	0.181 (*P* = 0.002)	0.207 (*P* = 0.002)
Satisfaction/quality	0.183 (*P* < 0.001)	0.222 (*P* < 0.001)	0.179 (*P* = 0.003)	0.233 (*P* < 0.001)
Sleep latency	0.072 (*P* = 0.069)	0.061 (*P* = 0.125)	0.137 (*P* = 0.022)	0.147 (*P* = 0.028)
Sleep efficiency	0.068 (*P* = 0.086)	0.053 (*P* = 0.182)	0.085 (*P* = 0.160)	0.073 (*P* = 0.277)
Sleep disturbances	0.135 (*P* < 0.001)	0.148 (*P* < 0.001)	0.091 (*P* = 0.129)	0.118 (*P* = 0.078)
Daytime dysfunction	0.079 (*P* = 0.044)	0.108 (*P* = 0.006)	0.039 (*P* = 0.518)	0.075 (*P* = 0.263)
Sleep duration	0.105 (*P* = 0.008)	0.067 (*P* = 0.089)	0.239 (*P* < 0.001)	0.169 (*P* = 0.011)

PSQI, Pittsburgh Sleep Quality Index; VMS, vasomotor symptom.

In multivariable analysis to examine the effect of treatment on change in sleep quality while controlling for changes in VMS, the difference in average improvement in PSQI global score between treatments, though attenuated, remained significant after adjustment for average improvement in each symptom (*P* = 0.020 adjusting for changes in hot flashes; *P* = 0.004 adjusting for changes in night sweats). Controlling for the effects of treatment, the associations between the change in each VMS and change in sleep remained significant as well (*P* = 0.002 for hot flashes and *P* = 0.029 for night sweats).

### Post hoc VMS subgroup analysis

At baseline, 226 women reported having moderate to severe night sweats, and 279 women (some overlap with those having moderate to severe night sweats) reported having moderate to severe hot flashes. In these respective subgroups of women, there were significant and positive correlations of changes in the global PSQI score, with changes in severity of night sweats (*r*_s_ = 0.181, *P* = 0.002) and with changes in severity of hot flashes (*r*_s_ = 0.207, *P* = 0.002; Table 3). In contrast to the results obtained on the overall set of participants, improvement in sleep latency in these women with moderate/severe symptoms correlated significantly with reduced severity of hot flashes (*r*_s_ = 0.137, *P* = 0.022) and night sweats (*r*_s_ = 0.147, *P* = 0.028); in addition, sleep duration correlated more strongly with reduced severity of hot flashes (*r*_s_ = 0.239, *P* < 0.001) and night sweats (*r*_s_ = 0.169, *P* = 0.011). In models adjusting for HT, the association between average changes in VMS and the average change in PSQI global score remained significant in both VMS subgroups (both *P* < 0.001). In contrast, the association of treatment with average change in PSQI global score was attenuated and no longer significant in both subgroups after adjustment for the average change in the corresponding VMS (*P* = 0.525 and 0.128 from partial tests of treatment effects in the subgroup with baseline hot flashes and with baseline night flashes, respectively).

## DISCUSSION

In a population of a majority of white recently menopausal women, improvements in sleep quality were observed with the use of low-dose HT (oral and transdermal) over a 4-year period. The global sleep score indicative of sleep quality in women at baseline is consistent with what has been reported for population-based studies.^2^ The average change in the global sleep score during treatment was a reduction of about 1.3 points in both groups randomized to HT. The magnitude of change in sleep score with HT was about twice as great as that reported by the PBO group (0.06), which is consistent with effects of HT on sleep reported in other studies.^12^

A second finding of the present study is that changes in sleep quality correlated with changes in VMS (hot flashes and night sweats)—a finding that persisted after controlling for treatment assignment to HT and consistent with other studies.^10^ However, a multivariable analysis on the overall set of women demonstrated significant partial effects of both treatment and change in VMS, indicating the alleviation of VMS does not fully account for the improved sleep outcomes among those assigned to HT and suggesting that HT affects other mechanisms associated with sleep. A causal relationship between these factors is hard to establish, as there is a bidirectional relationship of perceived sleep quality and VMS, in that poor sleep quality is both a consequence of VMS and also an influence on the extent to which VMS are perceived as bothersome. Some insight into why HT may affect sleep through mechanisms other than alleviation of VMS is provided by the subanalysis of the relationship between symptom relief and sleep domains in women reporting moderate/severe VMS at baseline. In these women, unlike the entire set of women, the association between the change in symptom severity and the change in PSQI score was attenuated and no longer significant after adjustment for treatment. These results suggest that in women with moderate to severe symptoms, but not in those with none to mild, the effects of HT on sleep are mediated through symptom relief—a finding consistent with conclusions of the recent meta-analysis of other studies of sleep, VMS, and HT.^12^

A third finding of the present study is the direct comparison between two formulations and doses of HT, which are commonly used in clinical practice, on sleep domains. The majority of previous sleep studies evaluated o-CEE at 0.625 mg/d.^12^ In the present study, following clinical guidelines that followed the cessation of the Women's Health Initiative in 2002 for use of lower doses of HT,^32^ 0.425 mg/d of o-CEE was used in KEEPS when it was designed in 2004. Although overall sleep quality was improved with HT, not all domains of sleep showed significant change averaged across the treatment period. The finding that t-E2 was more efficacious than o-CEE in alleviating sleep disturbances may be related to the pharmacokinetics of these two formulations. Transdermal E2 is likely to provide more consistent 24-hour estradiol dosing, whereas o-CEE may engender daytime peaks and night-time troughs if women took their o-CEE in the morning, thus, leading to less relief of sleep disturbances. In the entire group, sleep efficiency and sleep duration were not affected by either HT, reflecting, perhaps, that other factors such as life circumstances may impact these domains, especially in women who do not report moderate to severe symptoms.

Unlike what has been reported in other studies,^12^ daytime dysfunction was not improved by HT in the KEEPS cohort. This difference may be explained, in part, by a lower prevalence of women with poor sleep quality (global score >8) in the present study sample (24% at baseline) compared with other studies.^33,34^ In addition, the majority of participants in the KEEPS were white and recently menopausal as confirmed by strict criteria, whereas in other studies, women were more often of mixed ethnicity and were perimenopausal and also postmenopausal. The possibility that doses or formulations of HT directly influence specific domains of sleep apart from changes in VMS in women of different ethnicities and ages will require additional study.

There are difficulties in comparing self-reported sleep outcomes among clinical trials due to heterogeneity among the available questionnaires and the absence of objective testing.^12^ For example, self-reported sleep tools may include validated and nonvalidated scales, single-item or multiple-items measures, visual analog scales, and diaries such that clinical applicability of results in various reports to clinical management is uncertain.^35^ Yet, the use of sleep architecture measurements (polysomnography, wrist actigraphy) does not always correlate with perceived sleep quality,^36,37^ and due to costs and accessibility, these tests have limited applicability in large population settings. Therefore, self-report instruments remain crucial in the clinical assessment of outcomes after interventions to improve sleep. In studies of menopause, self-report instruments may allow clinicians to evaluate who might benefit from HT for sleep and who might benefit from additional clinical assessment, such as testing for sleep apnea. Although the PSQI contains questions regarding symptoms of snoring and stopping breathing during the night, the responses to these questions are included in an overall score for sleep disturbances and may not provide accurate information regarding sleep apnea for those individuals who sleep alone. However, the answers to these questions may provide information for the individual physician who examines the questionnaire for their individual patient.

This present study has a number of strengths. Evaluating the recently menopausal women enrolled in KEEPS allowed many of the deficiencies of the prior literature to be addressed. First, KEEPS was a randomized clinical trial enrolling a large number of well-characterized and otherwise healthy, recently menopausal women meeting the standard and stringent clinical and biochemical criteria for menopause. In addition, the design allowed for a direct comparison between the two modalities of menopausal HT commonly used in current clinical practice—o-CEE and t-E2. The differences in pharmacokinetics and dynamics between the products may help to direct clinical decisions related to which formulation best meets the women's needs. A second strength of the study is the use of a validated tool for self-reporting global sleep quality and domains of sleep. The PQSI provided important qualitative information on the domains of sleep that cannot be obtained by laboratory analysis with important implications for women whose major sleep complaints relate to their ability to fall asleep, sleep disturbances, and overall sleep satisfaction. Because data regarding VMS was by self-report, responses of symptom severity are susceptible to misclassification bias and may under or over-represent the symptoms. However, the associations between sleep and symptom severity are, to our knowledge, the first reported with use of low doses and two different routes of two different estrogen products in the same study. A limitation of the present study is that other stressors related to sleep quality such as marital, employment and socioeconomic status, allergies, caffeine intake, and numbers of children in the home were not considered. Although the study could be criticized for eliminating the domain of sleep medication, the use of such medications was low at baseline, limiting statistical power, and their use is not well-established with health outcomes.^15^ Additionally, no clinical assessments were obtained regarding other potential confounders of sleep such as emotional stress, obstructive sleep apnea, or restless leg syndrome. As with other studies, it is important not to generalize the results of this study to other groups not defined by the inclusion and exclusion criteria.

## CONCLUSIONS

In recently menopausal women, the overall sleep quality was improved by both HT regimens compared with PBO, with the transdermal estrogen formulation performing modestly better than the oral formulation. Of the studied sleep domains, sleep satisfaction, disturbances, and duration were improved, with sleep disturbances more improved by t-E2 than by o-CEE. Domains of sleep latency, efficiency, and daytime dysfunction were not affected by the HT regimes used in this study. Alleviation of VMS was associated with improvements in overall sleep quality. This result in not an unexpected outcome as it is common to use amelioration of VMS as a clinical guide to treatment. In a subset of women reporting moderate to severe VSM at baseline, reduced symptoms also correlated with improvement in the domain of sleep latency. These findings suggest that at least one way to approach the use of HT for sleep complaints is to assess the severity of VMS, and perhaps explore additional underlying problems affecting sleep, for example, obstructive sleep apnea.

Sleep disorders in midlife women warrant evaluation because treatment can lead to substantial improvements in quality of life and health outcomes.^1,17^ These results from a sufficiently powered, randomized clinical trial suggest that fostering a conversation about sleep quality, and sleep domains during clinical encounters may be a more appropriate guide for a patient-centered approach for achieving optimal sleep health.
